# S15-5 Executive and physical functions among community-dwelling older adults: results from the PASSWORD study

**DOI:** 10.1093/eurpub/ckad133.076

**Published:** 2023-09-11

**Authors:** Anna Tirkkonen, Timo Törmäkangas, Jenni Kulmala, Tuomo Hänninen, Anna Stigsdotter Neely, Sarianna Sipilä

**Affiliations:** University of Jyväskylä, Finland; University of Jyväskylä, Finland; Tampere University, Finland; Karolinska Institutet, Sweden; Finnish Institute for Health and Welfare, Finland; Kuopio University Hospital, Finland; Karlstad University, Sweden; Luleå University of Technology, Sweden; University of Jyväskylä, Finland

## Abstract

**Purpose:**

Cognitive and physical functions are key factors for safe walking. As these functions deteriorate with age walking may be compromised among older adults. However, cognitive and physical decline may be attenuated with cognitive and physical training. This study investigated the associations between cognitive, especially executive, and physical functions and sex differences in these associations in physically inactive older adults. Additionally, the role of participant characteristics in cognitive and physical training-induced change in executive functions was investigated

**Methods:**

314 older adults aged 70-85 were recruited and randomized to PTCT (N = 155) or PT (N = 159). PT included two supervised training sessions a week and home exercises. PTCT included PT and cognitive training. Measurements were organized at baseline, 6 and 12 months. Physical functions were assessed with 10-meter maximal walking speed, 6-minutes walking distance, dual-task cost in walking speed, habitual walking speed and Short Physical Performance Battery (SPPB). Core skills of executive functions were assessed with Stroop (inhibition), Trail Making Test B (set shifting) and Letter Verbal Fluency (updating). Training compliance was based on participation in supervised training sessions. The data was analyzed with multiple linear regression analyses and longitudinal two-group linear path models.

**Results:**

Mean age of the participants was 74.5. Results showed that Verbal Fluency test was positively associated with faster maximal and habitual walking speed (β = 0.272, p<0.001, β = 0.184, p = 0.009 respectively), longer 6-min walking distance (β = 0.242, p<0.001) and higher scores in SPPB (β = 0.234, p<0.001), additionally, TMT B-A was positively associated with higher scores in SPPB (β=-0.236, p<0.001). No significant sex-differences were found. Additionally, Stroop improved significantly more in women and participants in the low compliance subgroup who received PTCT compared to participant receiving PT (difference -8.758, p = 0.001 and difference in -8.405, p = 0.010 respectively) No other significant associations were observed.

**Conclusion:**

Executive and physical functions are positively associated in older adults. The association depends on the physical task and the executive subdomain. Physical and cognitive training improves older adults’ executive functions. Women and participants who only occasionally engaged in training may gain additional benefits for inhibition from physical and cognitive training compared to physical training.

**Funding:**

Academy of Finland (Grant no. 296843).

